# Kidney Transplant in Fabry Disease: A Revision of the Literature

**DOI:** 10.3390/medicina56060284

**Published:** 2020-06-10

**Authors:** Irene Capelli, Valeria Aiello, Lorenzo Gasperoni, Giorgia Comai, Valeria Corradetti, Matteo Ravaioli, Elena Biagini, Claudio Graziano, Gaetano La Manna

**Affiliations:** 1 Department of Experimental Diagnostic and Specialty Medicine (DIMES), Nephrology, Dialysis and Renal Transplant Unit, S. Orsola-Malpighi Hospital, University of Bologna, 40138 Bologna, Italy; irene.capelli4@unibo.it (I.C.); valeria.aiello6@unibo.it (V.A.); lorenzo.gasperoni3@unibo.it (L.G.); giorgia.comai@aosp.bo.it (G.C.); valeria.corradetti@aosp.bo.it (V.C.); 2 Department of General Surgery and Transplantation, S. Orsola—Malpighi Hospital, University of Bologna, 40138 Bologna, Italy; matteo.ravaioli@aosp.bo.it; 3 Institute of Cardiology, Department of Experimental, Diagnostic and Specialty Medicine, University of Bologna, S. Orsola-Malpighi University Hospital, 40138 Bologna, Italy; elena.biagini73@gmail.com; 4 Unit of Medical Genetics, S. Orsola-Malpighi Hospital, 40138 Bologna, Italy; claudio.graziano@unibo.it

**Keywords:** enzyme replacement therapy, Fabry disease, Fabry nephropathy, kidney transplant

## Abstract

Fabry disease is classified as a rare X-linked disease caused by a complete or partial defect of enzyme alpha-galactosidase, due to *GLA* gene mutations. This disorder leads to intracellular globotriaosylceramide (Gb3) deposition associated with increased Gb3 plasma levels. Most of the symptoms of the disease, involving kidneys, heart and nervous system, result from this progressive Gb3 deposition. The incidence is estimated in 1/50,000 to 1/117,000 in males. Fabry nephropathy begins with microalbuminuria and/or proteinuria, which, in the classic form, appear from childhood. Thus, a progressive decline of renal function can start at a young age, and evolve to kidney failure, requiring dialysis or renal transplantation. Enzyme replacement therapy (ERT), available since 2001 for Fabry disease, has been increasingly introduced into the clinical practice, with overall positive short-term and long-term effects in terms of ventricular hypertrophy and renal function. Kidney transplantation represents a relevant therapeutic option for Fabry nephropathy management, for patients reaching end-stage renal disease, but little is known about long-term outcomes, overall patient survival or the possible role of ERT after transplant. The purpose of this review is to analyze the literature on every aspect related to kidney transplantation in patients with Fabry nephropathy: from the analysis of transplant outcomes, to the likelihood of disease recurrence, up to the effects of ERT and its possible interference with immunosuppression.

## 1. Introduction

Fabry disease (FD) is an X-linked lysosomal inherited disorder, caused by deficient or absent activity of the enzyme alpha-galactosidase, caused by the mutation in the *GLA* gene. This enzyme defect leads to the progressive accumulation of lysosomal glycosfingolipids, particularly globotriaosylceramide (Gl-3). The α-galactosidase A (*GLA*) gene is involved in the disease by causing a marked reduction in alpha-galactosidase enzymatic activity, and the subsequent deposition of its major substrate, globotriaosylceramide (Gb3), in endothelial cells, cardiomyocytes, fibroblasts, nerve cells and renal cells (podocytes, glomerular, mesangial and tubular cells) [1,2].

The reported annual incidence is 1 in 476,000 in the general population, but this might largely underestimate the true prevalence, due to the wide spectrum of clinical phenotypes [3].

The prevalence of the classic FD form ranges between 1/8454 and 1/117,000 male live births [4].

In order to improve the detection of Fabry patients, many newborn screening tests are used in clinical practice, and they currently represent the best strategy for identifying patients with FD at an early stage and without clinical manifestations [5].

Newborn screening demonstrated an unexpectedly high prevalence of the disease, as high as 1 in ~3100 newborns in Italy [6], 1 in 1500 in Taiwan [7], and 1 in 6212 in Japan [5,6].

So far, over 1000 *GLA* mutations have been characterized in the chromosomal region Xq22.1 (point missense mutations, splicing alterations, deletions, translocations and complex gene rearrangements). Approximately 60% of them are missense mutations, resulting in single amino acid substitutions in the alpha-galactosidase protein [7,8].

The type of mutation might influence the clinical presentation of the disease, even if a genotype–phenotypes correlation is not clear-cut, and a significant phenotypic variability among individuals with the same pathogenic variant has been observed [9,10]. Two major clinical subtypes of FD are known: the classic and the late onset. The classic form occurs in males with less than 1% alpha-galactosidase activity, and it is caused by different types of rearrangements, splicing defects and missense or nonsense variants. On the other hand, male subjects with more than 1% alpha-galactosidase activity have missense or splicing variants, and show a “later-onset” or “non-classic” form. In the classic subtype, the patients have prominent vascular endothelial cell glycosphingolipid accumulations. Typically, the onset of severe acroparesthesia, angiokeratoma, hyperhidrosis, corneal and lenticular opacities occurs in childhood or adolescence. Renal and cardiac manifestations can appear afterwards, with the progression of the disease [11]. Conversely, in the subtype of the later onset, the patients show prevalent cardiac or renal involvement [9]. The typical signs are left ventricular hypertrophy, that usually develops in the fourth to eighth decade, and renal disease, characterized by the occurrence of proteinuria, linked with kidney function impairment and evolving to end-stage renal disease (ESRD), but without acroparesthesias and angiokeratoma [12]. Moreover, the clinical manifestations in heterozygous females range from being asymptomatic throughout their whole life, to being as severe as affected males. This is accredited in part to random X-chromosomal inactivation (“lyonization”), that takes place in somatic cells during the embryonic development. Such a process is tissue-specific, and severely affected females are more likely to express the X chromosome with the *GLA* pathogenic variant in the organs in question [13].

The first signs of Fabry nephropathy in males with the classic phenotype usually arise between 10 and 20 years of age, and they are represented by glomerular hyperfiltration associated with mesangial cells proliferation/expansion at kidney biopsy. Glycolipid deposits are also present in the tubular epithelial cells, particularly of the distal nephron, arterial or arteriolar endothelial and interstitial cells, which are associated with an early concentrating defect. Concomitantly, the onset of microalbuminuria and proteinuria results from a glycolipid deposit in glomerular cells (podocytes), and also in mesangial cells and in endothelial cells, with subsequent basal membrane thickening and glomerulosclerosis. At around 30–40 years, when glomerular sclerosis exceeds 50% and tubulointerstitial damage progresses, renal failure appears, in many cases evolving to ESRD in successive decades. Given the presence of affected females and late-onset mutations, the range of nephropathy presentation and evolution is wide, with 50% of male patients at the age of 35 years and 100% at the age of 52 years [2,14].

The rate of decline in filtering capacity is about 12.2 mL/min per year in male patients with the classic phenotype, leading to a rapid progression towards ESRD [15,16]. In the late onset variant and in female patients, the decline of renal function is usually slower and less predictable [2,9,10].

Since 2001, enzyme replacement therapy (ERT) for FD has been increasingly introduced in the clinical practice, with positive short-term and long-term effects. ERT, with either Agalsidase alfa or Agalsidase beta, has been shown to be effective in the control of Fabry nephropathy progression [17,18,19]. Better outcomes may be observed when treatment is started at an early age, prior to the development of organ damage. Nevertheless, many FD patients still progress to ESRD, and they need organ transplants: the possible causes are a prolonged treatment time, a delayed beginning of therapy and/or a late diagnosis.

The present review is focused on kidney transplants in Fabry patients, and on the role of ERT after kidney transplantation.

## 2. Organ Transplantation as a Replacement Therapy for Enzyme Deficiency: An Ineffective Solution

At the beginning of the 1970s, FD was known as an inborn glycosphingolipid catabolism defect, causing its accumulation principally in the fibromuscular cells in the vessel wall, together with a decrease in alpha-galactosidase plasma levels [1,2]. In 1972, Philippart et al. reported a case of a 38-year-old man with FD, who received a deceased-donor kidney transplant. After surgery, his plasma alpha-galactosidase levels, which were untraceable before transplantation, increased from 5% to 20%, with an improvement of some clinical parameters (reduction of fatigability, complete resolution in terms of cramps and pain). This early evidence gave birth to the intriguing idea that kidney transplantation, besides its role in optimal renal function replacement, could also represent an effective enzymatic substitution strategy. In order to demonstrate this hypothesis, Clarke et al. performed serial measurements of galactosyl glucosylceramide (CTH) and its precursor, N-acetilgactosyl glucosylceramide (CAH), on blood and urine specimens from transplanted patients. Both CTH and CAH concentrations decreased respectively after kidney transplantation (CTH from 0.76 to 0.48 Umol per 100 mL, and CAH from 0.23 to 0.07 Umol per 100 mL), suggesting that CTH and CAH levels change after transplant as a result of the decrease of lipid rate formation, rather than increased catabolism by the graft. Indeed, these data did not support the concept that a kidney transplant could act as an enzymatic therapy [20,21].

Other attempts to replace the enzyme deficiency by transplantation have been reported. In 1979, Touraine et al. described the cases of two patients with FD and minimal renal involvement, treated with a transplant of fetal liver cells. The authors found a symptomatic improvement without plasmatic alpha-galactosidase level modification [22]. Recently, Likhitsup et al. described the case of a 52-year-old woman with alcoholic cirrhosis and kidney failure secondary to FD, documented at kidney biopsy, who received a combined liver and kidney transplant. At 4 weeks after transplant, her serum alpha-galactosidase level was unexpectedly normalized, but this condition was transient. The authors suggested that the temporary alpha-galactosidase normalization after combined liver and kidney transplantation was probably related to a short-term lysosomal enzyme release from the transplanted organ. In consequence, the patient later was treated with ERT for the known enzymatic deficit, linked to the underlying disease [23].

This discouraging result can be explained by the fact that in FD, there is no cross-correction of the enzymatic defect between cells, thus transplantation does not seem to amend the defect in other organs.

Hence, liver transplant, that is also considered a curative treatment in some enzymatic disorders with enzymatic deficiency expressed exclusively in the liver (as for ornithine transcarbamylase deficiency), does not appear to have long-term positive effects on alpha-galactosidase levels, or effectively reduce LysoGB3 levels in patients with FD [24].

## 3. Graft and Patient Outcomes in Fabry Disease Patients after Kidney Transplant

Since 1967, when the first kidney transplant in a patient with FD was performed, several concerns have been raised about such an approach, due to the high rates of infectious complications and early transplant failures. Several articles were published in the following years on this issue.

In 1975, a report from the ASC/NIH (American College of Surgeons and the National Institutes of Health) Renal Transplant Registry studied the role of renal transplant in several congenital and metabolic diseases, namely Alport syndrome, amyloidosis, cystinosis, diabetes mellitus, FD, familial nephritis, gout, medullary cystic disease and oxalosis. The results highlighted good outcomes for renal transplantation in most disorders, with the exception of FD and oxalosis. For FD, the graft survival after one year was 33% (only three patients of nine transplanted had functioning kidneys), and no evidence of relapse after graft was mentioned [25].

Later in 1981, a review by Maizel et al. describing a 10-year experience of a kidney transplant in FD, reported a patient survival rate of 26% at 5 years, and a high incidence of death from sepsis (4/8) [26].

For this reason, kidney transplants in FD patients were initially discouraged. Ten years later, promising data for graft and patient survival were presented from European and American registry analysis. From the European Dialysis and Transplant Association/European Renal Association Registry, 33 patients with FD enrolled in a 3-year observational study, and presented a graft survival comparable to other kidney diseases: 72% (24/33 graft) vs. 69% (22/33 graft). Moreover, the patient survival rate after kidney transplant was comparable to that of the group aged under 55 with non-Fabry nephropathies, respectively 84% (27 patients) vs. 87% (28 patients) [27]. A cohort study on 93 patients, from 1988 to 1998, by the US Renal Data System Registry reported a 1- and 5-year graft survival of 91% (83/93 graft) and 76% (70/93 graft), respectively, and a 5-year patient survival of 83% (77 patients), with no significant differences from the control population [28].

Finally, a retrospective monocentric study by Inderbitzin et al. aimed to evaluate kidney transplant effects on the long-term outcomes of 10 patients with end-stage renal failure due to FD, who received a transplant at a median age of 36 years. The authors found a graft survival rate of 90% at 5 years (9/10 graft), and 66% at 10 years (6/10 graft), and a patient survival rate of 100% at 5 years (10/10 patients) and 76% at 10 years (7/10 patients) [16].

As for very long-term outcomes, the retrospective cohort study, published in 2018 by Ersözlü et al. which includes follow-ups of Fabry kidney transplant grafts up to 25 years, has currently the longest reported experience on this subject. A total of 17 FD patients were enrolled, all of them kidney transplant recipients; 11 from deceased donors, 6 from living donors. The authors reported a 10- and 25-year graft survival rate of 92% (15 patients) and 22% (4 patients), respectively, and a 10- and 25-years patient survival rate of 100% (17 patients) and 25% (4 patients), respectively. Moreover, the study found a better death-censored graft survival rate in FD patients compared with non-FD matched controls (*p* = 0.03), indicating that Fabry kidney transplant recipients had a higher mortality as compared to non-Fabry controls, for cardiac events with functioning grafts (median age at death, 59 years) [29]. Long patient survival was comparable even with Fabry patients who underwent dialysis approximately 20 to 25 years ago, from the European Registry. Further, in this case, transplant represents the best option (90% versus 41%), according to Tsakiris et al. [27]. All these studies indeed suggest that kidney transplant outcomes in Fabry patients, defined as graft survival and patient survival, are similar to those of patients transplanted for other nephropathies. Therefore, nowadays kidney transplantation really represents a relevant therapeutic option for the management of Fabry nephropathy.

## 4. Enzyme Replacement Therapy Impact on Kidney Transplant Outcomes and Immunosuppressive Therapy

The introduction of ERT to counteract the metabolic defect in Fabry disease dates back to 2000s. Both the currently available drugs, Agalsidase alfa (Replagal(^®^), Shire Human Genetic Therapies AB, Lund, Sweden) and Agalsidase beta (Fabrazyme^®^, Genzyme, a Sanofi company, Cambridge, MA, USA) [24,25], must be administered intravenously with different regimens [9,29,30,31]. Data on ERT efficacy in kidney transplants are limited. In 2004, Mignani et al. performed a single center pilot study that showed that ERT (Agalsidase beta, administered every 2 weeks for 18 months, in three kidney transplant recipients) was safe and protective against extrarenal manifestation in kidney transplant recipients with FD. Concerning safety, the authors did not observe any treatment-related adverse events, intolerance episodes, or seroconversions in transplanted patients with stable graft function and with no need for immunosuppression regimen adjustment, treated with total 116 intravenous infusions. About efficacy, some patients experienced extreme pain that disappeared within 2 months after the manifestation. Cardiac ultrasound revealed an amelioration in 2 out of the 3 patients receiving all the planned infusions for left ventricular mass, end diastolic diameter and cardiac contractility, expressed by ejection fraction. The third patient, who had a 5-month interruption in their ERT therapy, kept a stable diastolic diameter and ejection fraction, but displayed some progressive cardiac morphologic abnormalities. All the patients showed persisting mild mitral insufficiency, and the affected individual also showed atrial fibrillation [32]. In 2008, the same group conducted a survey of 34 FD patients on ERT, 17 of them on dialysis (1 no ERT, 7 with ERT after beginning of hemodialysis; mean 1.1 years) and 17 kidney transplant recipients (15 with ERT after kidney transplant, mean 4.8 years). The overall average ERT follow-up was 45.1 (hemodialysis) and 48.4 (transplant) months in the two groups. This study revealed that, in the subgroup of 17 kidney transplanted recipients, the rate of creatinine clearance decline in the patients on ERT was milder than in patients without ERT (−1.92 mL/min/year vs. 12 mL/min/year, respectively), suggesting an important protective effect of ERT on graft function [33].

Cybulla et al. studied the effect of Agalsidase alfa in 20 kidney transplant recipients collected by FOS (Fabry Outcome Survey) registry data analysis (27 patients; 20 patients on ERT compared to 7 patients not on ERT). Five patients received ERT before transplantation. After an average of 3.5 years of treatment, ERT patients showed a slight decrease in renal function (59.2 mL/min/1.73 m^2^ at baseline vs. 51.1 mL/min/1.73 m^2^), and stable proteinuria [34]. It should be underlined that the positive effect in renal function may be related to the presence of normal enzyme activity within the graft. Regarding the extrarenal outcomes, cardiovascular disease represents one of the main causes of morbidity and mortality after renal transplantation in non-FD patients [35], with a reported post-transplant incidence of new onset chronic atrial fibrillation of 3.5% and 7.3%, at 12 and 36 months, respectively [36].

According to the mentioned studies, ERT is also able to delay the progression of left ventricular hypertrophy and reduce cerebrovascular events in patients with FD undergoing kidney transplants [32,33,34]. In the study by Mignani and colleagues mentioned before (34 Fabry patients; 17 on dialysis and 17 kidney transplant recipients), additional arrhythmia episodes were recorded, although no data are provided on patient survival [32]. A similar trend was observed for cerebrovascular events, with 35% less cases in transplanted patients (nine acute cerebrovascular events in the dialyzed population versus three events in the transplanted population) [33]. After 3 years of ERT, the left ventricular max index showed a rise of 19% (from 210.9 g/m^2^ to 251.0 g/m^2^) in the seven dialysis patients, while it was reduced by 6% (from 234.6 g/m^2^ to 220.8 g/m^2^) in the transplanted patients [32]. In the second study by the group of Cybulla on 20 kidney transplant recipients with FD, left ventricular hypertrophy was increased in patients without therapy (n = 4), compared to those on ERT (n = 9) (86.5 g/m^2^ vs. 64.9 g/m^2^; *p* = n.s.) [34].

The main studies comparing graft and patient survival rates in kidney transplant recipients, with Fabry and non-Fabry Disease as primary nephropathy, are reported in Table 1.

In August 2018, the FDA approved chaperon therapy with migalastat (Galafold™, Amicus Therapeutics, Cranbury, NJ, USA) as an alternative to ERT in FD. These agents are analogs of the terminal galactose of GL-3, which selectively and reversibly bind and stabilize some mutant forms of alpha-galactosidase. This linkage prevents the misfolding of the protein and promotes cellular trafficking to lysosomes. Therefore, Migalastat therapy is only feasible for patients with amenable mutations [37]. In contrast to ERT, Migalastat is orally available and has broad tissue spread. Currently, Migalastat is not recommended in patients with GFR < 30 mL/min [38]. No limitations are mentioned regarding kidney transplant recipients. The data from safety studies indicate that Migalastat is well tolerated in FD patient, but no study analyzed the effect of this therapy after kidney transplantation.

In summary, ERT is considered safe after kidney transplantation, and protective in terms of graft and patient survival, continuing even after the transplant failure to carry out a protective action on the extra-renal aspects of the disease.

As a consequence of the rarity of the pathology, the literature is mainly based on retrospective studies, often with small and non-homogeneous cohorts. The currently available evidence shows univocally short- and long-term graft and patient outcomes not inferior to those of ESRD non-Fabry patients. Based on these data, in 2013, indications for FD treatment were published by European Renal Best Practice; kidney transplantation is recommended as a valuable option in patients who are eligible for this intervention. Moreover, after transplantation, ERT is suggested for extra-renal indications [39].

## 5. Effect of Immunosuppression on ERT

ERT infusions may lead to two types of immune reactions: an acute infusion-associated reaction (IAR), and a long-term effect of therapy inhibition due to antibody formation. This antibody-mediated inhibition is more frequent in the classical FD phenotype, and it is associated with higher plasma lyso-Gb3 concentrations, increased left ventricular mass, and a progressive renal function decline [40,41,42].

In 2018, Lenders et al. published a retrospective study to clarify the role of immunosuppressive therapy on ERT inhibition in FD patients. A total of 26 FD transplanted patients (24 kidneys and 2 heart) under immunosuppressive regimen, aged 51 ± 11 years, were enrolled for the study with a follow-up of 80 ± 72 months. This cohort was divided into two groups: ERT-naïve group (6 patients transplanted before 2001, and 2 patients’ late referral necessitating organ transplantation and ERT together) and ERT group (18 patients that started ERT before transplant). No patient of the first group (n = 8) developed antibodies within the follow-up (80 ± 72 months) following ERT initiation. The prevalence of increased serum-mediated ERT inhibition was 40% in the second group, and it was not selective for any recombinant *GLA* product. Patients with anti-ERT antibodies showed a significantly increased risk of developing FD-typical symptoms; mostly impaired cardiac and renal function. In transplant recipients under an initial immunosuppressive regimen (prednisolone, tacrolimus and mycophenolate-mofetil/-acid), ERT inhibition was found to be decreased after transplantation (n = 12; *p* = 0.0160). Tapering of immunosuppression (mainly prednisolone) is linked to increased levels of anti-ERT antibodies (n = 4, median (range): 16.6%; (6.9; 36.9%); *p* = 0.0972) over time. In conclusion, immunosuppressive therapy in transplant recipients appears to prevent de novo ERT inhibition in ERT-naïve patients, while for patients on stable ERT before the transplant, ERT inhibition seems temporarily suppressed. High-dose immunosuppression resulted in antibody level reduction, without improving long-term outcomes [42].

## 6. Recurrence of Fabry Disease in Kidney Transplantation: The Histological Findings

In the literature, data about the histological recurrence of Fabry in transplanted organs are inconsistent: all the studies are retrospective and with different timings of transplants, biopsies and ERT commencement.

From 1972 to 1998 (before the ERT era), 11 case reports with histological data from kidney transplants were published. Five biopsies where the autoptic and the timing of the biopsy was various: in 2 cases, data refer to less than one year (6–17 months); in 4 cases, 5 years; in another 2 cases, 10 years; and in 3 cases, longer than 10 years. No information is mentioned about the *GLA* mutation involved. Histology shows typical endothelial, interstitial and glomerular deposits (multilamellar bodies, sphingolipid inclusions) in 6 to 11 cases (Table 2) [21,43,44,45,46,47,48,49,50,51]. It was not clear whether the occurrence of the ceramide accumulation in the renal graft could add further risk of graft failure.

A study by Ersözlü et al. on 20 graft biopsies from 11 kidney transplant patients found glycosphingolipid deposits (lamellar lysosomal inclusions inside vascular endothelial cells) only in two brothers. The biopsy was performed in the elder brother 13.8 years post-kidney transplant (pre-ERT era), and in the younger brother 22.7 years after kidney transplant, and he started ERT at 14.3 years after the transplant. In contrast to these patients, two other patients, who also had been not under ERT for a long time (10 and 11.6 years), and biopsied after 11.6 and 21.8 years, had no lysosomal inclusions under light and electron microscopy. It is not possible to find a direct correlation between ERT and glycosphingolipid accumulation; moreover, not all patients with no ERT after transplantation, even for a long time, showed a glycosphingolipids stockpile [29].

In conclusion, studies published up to now do not exclude typical accumulations of glycosphingolipids recurring in the endothelial cells of the transplanted kidney, even in patients on ERT. It should be emphasized that the studies are all retrospective, and with quite different biopsy and transplant timings. The presence of glycosphingolipid deposits in kidney transplants does not seem to affect the outcome, in patients either with or without ERT, nor has clinical FD relapse been reported after kidney transplantation.

**Figure 1:**
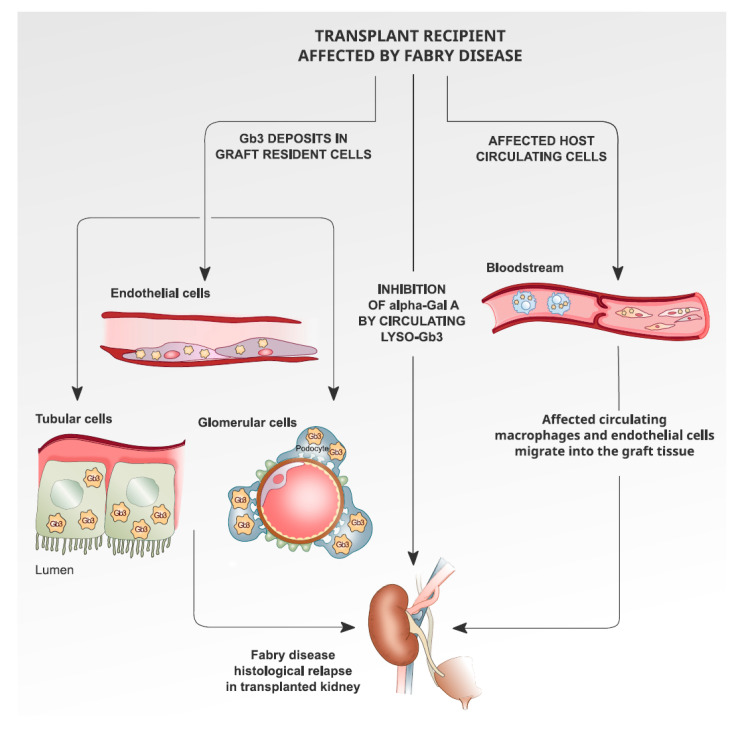
Possible mechanisms that can cause histological recurrence of Fabry nephropathy in the transplanted organ.

In Figure 1, we propose the description of the possible mechanisms that can cause histological recurrence of Fabry nephropathy in the transplanted organ.

## 7. Conclusions

Kidney transplantation represents the gold standard for organ substitution in eligible patients with FD. Graft survivals are comparable to non-Fabry disease patients, and long-term graft survival may be reduced by cardiovascular morbidity and mortality. Clinical relapse of Fabry nephropathy after transplant is not reported, even if histological recurrence may not be excluded; the latter does not seem to have an impact on the outcome of the graft, even in the long term. Organ transplant does not emerge as an effective enzymatic substitution in Fabry patients, so ERT therapy is recommendable in kidney transplant recipients for extra-renal symptoms. At present, no studies have investigated Migalastat in kidney transplant recipients. Immunosuppressive therapy seems to prevent de novo ERT inhibition in ERT-naïve patients, and to reduce ERT antibody level reduction, although this finding does not confer long-term protection or improve outcomes.

## Figures and Tables

**Table 1:** Compared graft and patient survival in Fabry and non-Fabry Disease kidney transplant recipients. The years of follow up of each study are detailed in brackets.

Compared Outcomes					
	Pre ERT Era		ERT Era		Non Fabry
	Ojo et al., (1988–1998)	Inderbitzin et al., (1964–1998)	Mignani et al., (2008)	Ersolozlu et al., (1979–2017)	USRDS (2006–2011)
5 years graft	76%	90%	87.5%	93%	75%
5 years patients	83%	100%	100%	100%	85%
10 years graft	56%	66%		92%	48%
10 years patients	67%	76%		100%	64%
25 years graft				22%	
25 years patients				25%	

ERT: enzyme replacement therapy; USRDS: United States Renal Data System.

**Table 2:** Most relevant studies on histology findings pertaining to kidney transplant recipients with Fabry disease.

Year of Publication	Authors	Number of Patients	Biopsy Timing	Histology Findings
1972	Clarke et al.	1	>1 year	Glycolipids deposition
1981	Farragiana et al.	1	>1 year	Glomerular, tubular and interstitial deposits
1973	Buhler et al.	1	5 years	No deposits
1982	Clement et al.	1	5 years	No deposits
1986	McMahon et al.	1	5 years	Endothelial depositions
1987	Popli et al.	1	5 years	Zebra bodies
1998	Erten et al.	1	5–10 years	No deposits
1987	Friedaender et al. [52]	1	5–10 years	No deposits
1982	Bannwart et al. [53]	1	<10 years	No deposits
1991	Mosnier et al.	1	<10 years	No deposits
1995	Gantenbein et al.	1	<10 years	Tubular, interstitial deposits
2018	Ersözlü et al.	17	<25 years	Glomerular, tubular and interstitial deposits in 2 patients
